# Analysis of risk factors for determining the need for prostate biopsy in patients with negative MRI

**DOI:** 10.1038/s41598-021-83802-z

**Published:** 2021-03-15

**Authors:** Linghui Liang, Feng Qi, Yifei Cheng, Lei Zhang, Dongliang Cao, Gong Cheng, Lixin Hua

**Affiliations:** Department of Urology, The First Affiliated Hospital of Nanjing Medical University, 300 Guangzhou Road, Nanjing, 210029 Jiangsu People’s Republic of China

**Keywords:** Cancer, Urology, Energy science and technology

## Abstract

To analyze the clinical characteristics of patients with negative biparametric magnetic resonance imaging (bpMRI) who didn’t need prostate biopsies (PBs). A total of 1,012 male patients who underwent PBs in the First Affiliated Hospital of Nanjing Medical University from March 2018 to November 2019, of 225 had prebiopsy negative bpMRI (defined as Prostate Imaging Reporting and Data System (PI-RADS 2.1) score less than 3). The detection efficiency of clinically significant prostate cancer (CSPCa) was assessed according to age, digital rectal examination (DRE), prostate volume (PV) on bpMRI, prostate-specific antigen (PSA) and PSA density (PSAD). The definition of CSPCa for Gleason score > 6. Univariate and multivariable logistic regression analysis were used to identify predictive factors of absent CSPCa on PBs. Moreover, absent CSPCa contained clinically insignificant prostate cancer (CIPCa) and benign result. The detection rates of present prostate cancer (PCa) and CSPCa were 27.11% and 16.44%, respectively. Patients who were diagnosed as CSPCa had an older age (*P* < 0.001), suspicious DRE (*P* < 0.001), a smaller PV (*P* < 0.001), higher PSA value (*P* = 0.008) and higher PSAD (*P* < 0.001) compared to the CIPCa group and benign result group. PSAD < 0.15 ng/ml/cm^3^ (*P* = 0.004) and suspicious DRE (*P* < 0.001) were independent predictors of absent CSPCa on BPs. The negative forecast value of bpMRI for BP detection of CSPCa increased with decreasing PSAD, mainly in patients with naive PB (*P* < 0.001) but not in prior negative PB patients. 25.33% of the men had the combination of negative bpMRI, PSAD < 0.15 ng/ml/cm^3^ and PB naive, and none had CSPCa on repeat PBs. The incidence of PB was determined, CSPCa was 1.59%, 0% and 16.67% in patients with negative bpMRI and PSAD < 0.15 ng/ml/cm^3^, patients with negative bpMRI, PSAD < 0.15 ng/ml/cm^3^ and biopsy naive and patients with negative bpMRI, PSAD < 0.15 ng/ml/cm^3^ and prior negative PB, separately. We found that a part of patients with negative bpMRI, a younger age, no suspicious DRE and PSAD < 0.15 ng/ml/cm^3^ may securely avoid PBs. Conversely PB should be considered in patients regardless of negative bpMRI, especially who with a greater age, obviously suspicious DRE, significantly increased PSA value, a significantly small PV on MRI and PSAD > 0.15 ng/ml/cm^3^.

## Introduction

Multi-parametric magnetic resonance imaging (mpMRI) has marvelously sensitive power in detecting clinically significant prostate cancer (CSPCa)^1^. The function of mpMRI in prostate cancer (PCa) management has been continuously growing in the past decade^2^. The current standard sequences are a combination of T2 weighted imaging (T2WI) plus two functional MRI sequences: Diffusion Weighted Imaging (DWI) and Dynamic Contrast Enhanced (DCE) imaging^3^. Recent studies elaborated bi-parametric MRI (bpMRI) is a feasible implement with which to distinguish CSPCa. They recommended using supplemental implements to increase PCa detection in patients with Prostate Imaging Reporting and Data System (PI-RADS v2.1) ≥ 3^4^. In addition, bpMRI has been displayed to have a diagnostic accuracy and PCa detection rate that are equivalent to those of mpMRI. BpMRI has the potential to result in substantial cost benefit and increased access to MRI in the diagnostic workflow and risk-stratification of men being evaluated for PCa when compared to conventional mpMRI^5^. But there are still some debates about the interpretation of negative MRI. It is still controversial whether prostate biopsies (PBs) are necessary for patients without suspected index lesions on bpMRI. In this case, because there is no targeted lesion, targeted biopsy is not applicable^6^. Repeat biopsies were usually needed to establish the diagnosis, but they can lead to over-detection and over-treatment of clinically insignificant prostate cancers (CIPCa), with a limited detection rate^7^. It has been implied that added negative predictors of CSPCa can help refine the decision making algorithm to more self-confidently determine whether patients with negative bpMRI need to undergo PBs^8^.

We analyzed the retrospective data of a group of patients with prebiopsy negative MRI in order to determine the factors that influence the detection rate of PCa or CSPCa, with which we can establish a low risk of significant disease in patients with negative MRI. Hence biopsy can be avoided in this group and further clinical decisions for this population can also be provided.

## Materials and methods

### Study design and population

**Figure 1:**
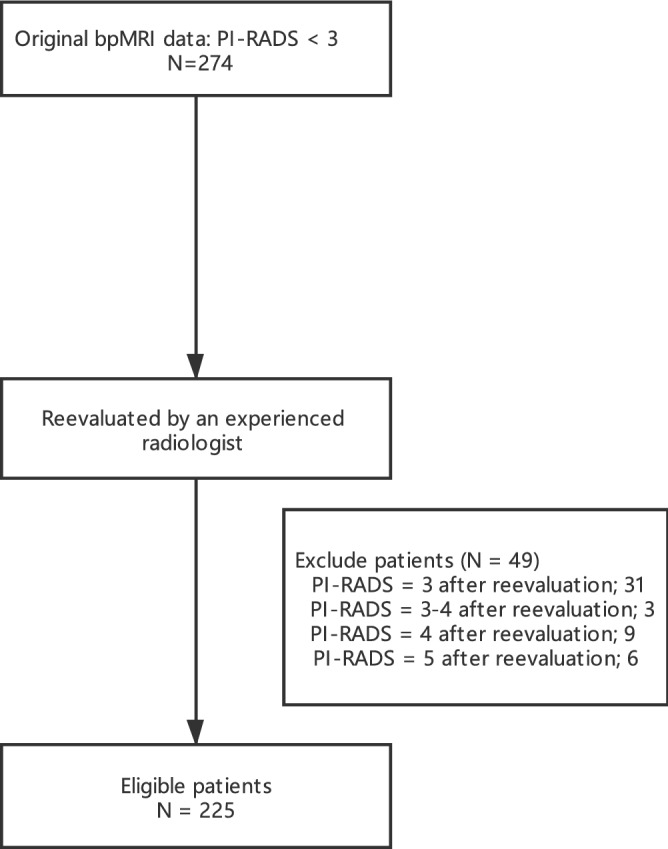
Schematic tree of study group.

We identified consecutive male patients who underwent prebiopsy bpMRI at our institution from March 2018 to November 2019. Patients were included in study if they had negative bpMRI, defined as PI-RADS v2.1 score less than 3^9^. During this period, 274 patients had prebiopsy negative bpMRI. Then we invited an experienced radiology doctor to reevaluate the MRI images of these patients. Forty-nine patients were excluded because they were reassessed as nonnegative MRI (Fig. 1). Finally, we analyzed 225 patients with negative MRI who met the inclusion criteria. All of the enrolled patients underwent systematic 14-core biopsy. All patients signed informed consent before conducting prostate biopsy. Rates of cancer detection were calculated from this group. CSPCa was defined as International Society of Urological Pathology (ISUP) grade 2 or higher based on histopathology findings, and scored as Gleason score (GS) 3 + 4 or higher^10^. This study was approved by the institutional review board and the First Affiliated Hospital of Nanjing Medical University Ethics Committee and informed consent was obtained from all patients and volunteers. We obey the principles of the 1983 Declaration of Helsinki. In other words, all of experiments in this paper obey this principle.

### BpMRI imaging protocol

BpMRI Imaging was performed on a 3 T MRI system (Verio, Siemens, Erlangen, Germany). The MRI acquisition protocol included high resolution T2-weighted, diffusion-weighted and parametric apparent diffusion coefficient maps were calculated from the DWI^6^.

### BpMRI interpretation and demarcation of MRI suspicious lesion (mSL)

A single radiologist specializing in prostate imaging evaluated all examinations based on previously published prostate MRI interpretation and reporting methodology^11^. No mSLs were scored 1 or 2 and included in the evaluation^6^. If there are two suspicious lesions, PI-RADS takes the maximum value.

### Biopsy procedure

Ultrasonographt system (HD11 XE, Philips Ultrasound, Inc. 22,100 Bothell Everett Highway Bothell, WA 98,021–8421-8431, USA), head scanning probe, disposable biopsy gun and 18-G needles (MC1820, Bard Peripheral Vascular, Inc. 1625 West 3rd Street, Tempe, Arizona 85,281, USA) were used for biopsies.

Transrectal ultrasound (TRUS) guided transrectal PB was performed by 5 experienced urologists with the patient without local anesthesia in operation room. Systematic extended sextant 12-core biopsies and 2-core biopsy of the apex region of prostate were obtained with an 18-G needle and each core was independently labeled and submitted to histology in separate containers.

### Statistical analysis

We evaluated and updated our prostate MRI protocol which meets the recommendation of PI-RADS v2.1. Prostate volume (PV) was calculated using the ellipsoid formula based on MRI measurement^12^. All bpMRI were evaluated by an experienced radiologist with a minimum of 10 years of experience with prostate MRI.

To analyze data the patients were divided into 3 groups, including CSPCa, clinically significant prostate cancer (CIPCa) and benign groups. To assess predictors of absent CSPCa on PB patients were compared as CSPCa vs CIPCa group and benign group. Receiver Operating Characteristic (ROC) curve analysis was used to detect absent CSPCa on PB. Multivariable logistic regression analysis was done with clinically relevant parameters and statistically significant variables on univariate analysis^8^.

Data was analyzed using SPSS software (version 22.0, IBM Corp., Armonk, NY, USA) and GraphPad Prism 8 software (version 8.2.1, GraphPad, San Diego, CA, USA). The Mann–Whitney U test, the Kruskal–Wallis and post hoc tests were used for continuous variables and the chi-square or Fisher exact test was used for categorical variables. Statistical significance was defined as *P* < 0.05. We excluded patients with bpMRI done elsewhere and those who had undergone any prior surgical or medical treatment for PCa or BPH. And MRI should be done within 3 months before PB.

## Results

Table 1 listed demographics. Of 208 (92.44%) patients were PB naive and 17 (7.56%) underwent previous negative PB. PB detected PCa in 61 men (27.11%) and CSPCa in 37 (16.44%).

**Table 1:** Demographic data of patients with negative MRI who underwent PB.

Variables	Overall	Biopsy outcome			*P* Value
		CSPCa	CIPCa	Benign	
No. pts	225	37	24	164	–
Median age (IQR)	66.0 (61.0–72.5)	71.0 (65.5–76.0)	70.5 (63.5–74.0)	65.0 (59.0–70.8)	< *0.001*
Median kg/m^2^ BMI (IQR)	24.15 (22.49–25.74)	23.94 (22.49–26.04)	24.45 (23.12–27.12)	24.15 (22.41–25.56)	*0.412*
Median ng/ml PSA (IQR)	9.38 (6.62–13.42)	12.60 (7.66–20.37)	9.00 (7.22–12.16)	9.02 (6.54–12.45)	*0.008*
Median cm^3^ PV (IQR)	43.35 (31.00–62.75)	30.05 (24.73–36.39)	45.67 (33.28–58.92)	47.68 (34.95–67.42)	< *0.001*
Median ng/ml/cm^3^ PSAD (IQR)	0.21 (0.14–0.34)	0.42 (0.32–0.59)	0.20 (0.15–0.26)	0.19 (0.12–0.28)	< *0.001*
PSAD ≥ 0.15 ng/ml/cm^3^ (%)	162 (72.00)	36 (97.30)	19 (79.17)	107 (65.24)	< *0.001*
PSAD < 0.15 ng/ml/cm^3^ (%)	63 (28.00)	1 (2.70)	5 (20.83)	57 (34.76)	
Suspicious DRE (%)	36 (16.00)	16 (43.24)	4 (16.67)	16 (9.76)	< *0.001*
**No. prostate biopsy status (%)**					
Naive	208 (92.44)	36 (97.30)	24 (100.00)	148 (90.24)	*0.157*
Previous neg	17 (7.56)	1 (2.70)	0 (0)	16 (9.76)	

*IQR* Interquartile range, *BMI* Body Mass Index, *PSA* prostate specific antigen, *PV* prostate volume on MRI, *PSAD* PSA density, *DRE* digital rectal examination, *CSPCa* clinically significant prostate cancer, *CIPCa* clinically insignificant prostate cancer.

**Figure 2:**
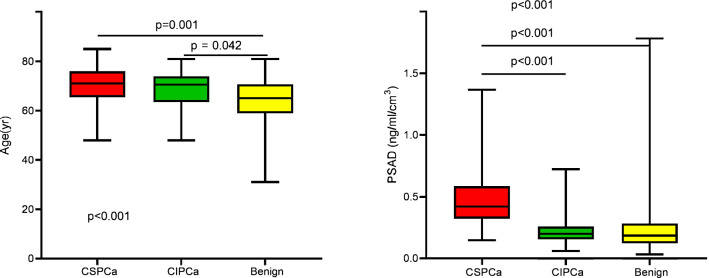
Age and PSAD by PBx histology in patients with negative MRI.

Contrast among CSPCa, CIPCa and benign groups showed that prostate-specific antigen (PSA), age, PV, PSA density (PSAD) and suspicious digital rectal examination (DRE) were statistically different among these groups (*P* = 0.008, remaining *P* < 0.001, Table 1, Fig. 2).

**Figure 3:**
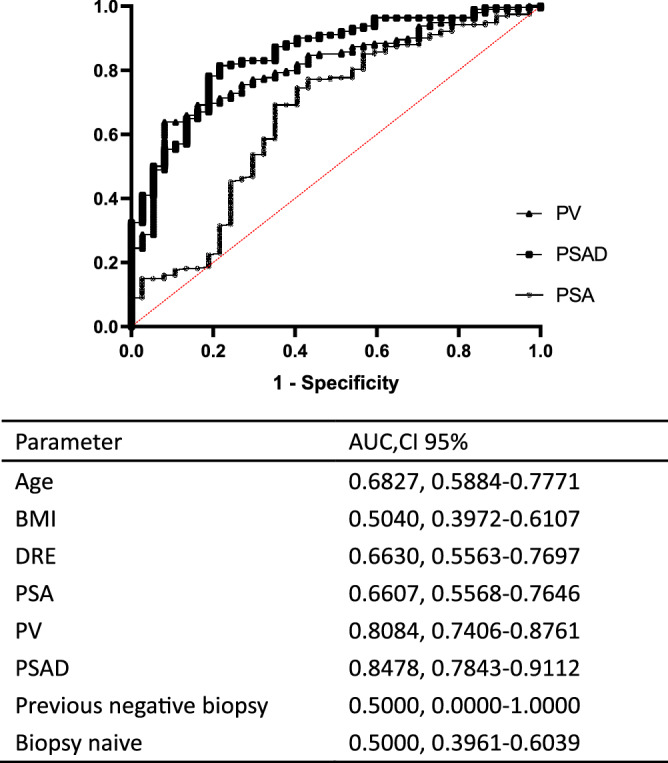
ROC curve analysis of absent CSPCa in patients with negative MRI.

Comparing 37 patients with CSPCa to 188 with absent CSPCa, univariate analysis showed that a younger age (*P* = 0.001), lower PSA value (*P* = 0.003), greater prostate volume (PV, *P* < 0.001), lower PSAD (*P* = 0.011), especially PSAD < 0.15 ng/ml/cm^3^ (*P* = 0.005) and suspicious DRE (*P* < 0.001) were predictors of detection of absent CSPCa in patients with negative MRI. Furthermore, multivariate analysis authenticated that a younger age (*P* = 0.001), greater PV (*P* < 0.001), lower PSAD (*P* = 0.011) and suspicious DRE (*P* = 0.006) were independent predictors of detection of absent CSPCa (Table 2). ROC curve analysis of absent CSPCa detection reveal a larger AUC for PSAD compared to PV or PSA or other parameters (0.8478 vs 0.8084 vs 0.6607, Fig. 3, Table 3). All 57 patients (25.33%) with PSAD less than 0.15 ng/ml/cm^3^ and naive biopsy history had no CSPCa on biopsy. Conversely 36 of the 151 patients (23.84%) with PSAD ≥ 0.15 ng/ml/cm^3^ and naive biopsy had CSPCa on biopsy (Table 3).

**Table 2:** Predictors of absent PB detected, CSPCa in men with negative MRI.

	Univariate		Multivariate	
	OR (95% CI)	*P* value	OR (95% CI)	*P* value
Age	0.918 (0.872–0.966)	*0.001*	0.902 (0.846–0.961)	*0.001*
PSA	0.941 (0.903–0.979)	*0.003*	*–*	–
PV	1.088 (1.050–1.127)	< *0.001*	1.083 (1.039–1.129)	< *0.001*
PSAD	0.006 (0.001–0.045)	< *0.001*	0.069 (0.009–0.547)	*0.011*
PSAD < 0.15 ng/ml/cm^3^	0.056 (0.008–0.421)	*0.005*	*–*	–
DRE suspicious for PCa (yes or no)	0.156 (0.070–0.347)	< *0.001*	0.256 (0.098–0.672)	*0.006*

*OR* odds ratio, *PSA* prostate specific antigen, *PV* prostate volume on MRI, *PSAD* PSA density, *DRE* digital rectal examination.

**Table 3:** MRI negative predictive value of CSPCa detection according to PSAD and PB history.

PSAD (ng/ml/cm^3^)	No. MRI Neg predictive value/total no. (%)		
	Overall	Biopsy Naive	Prior Neg Biopsy
0.15 or Greater	36/162 (77.78)	36/151 (76.16)	0/11 (100.00)
Less than 0.15	1/63 (98.41)	0/57 (100.00)	1/6 (83.33)
*P* value	< 0.001	< 0.001	0.353
0.15 or Greater	36/162 (77.78)	36/151 (76.16)	0/11 (100.00)
0.10-Less than 0.15	1/39 (97.44)	0/35 (100.00)	1/4 (75.00)
Less than 0.10	0/24 (100.00)	0/22 (100.00)	0/2 (100.00)
*P* value	< 0.001	< 0.001	0.447

*PSAD* PSA density.

**Figure 4:**
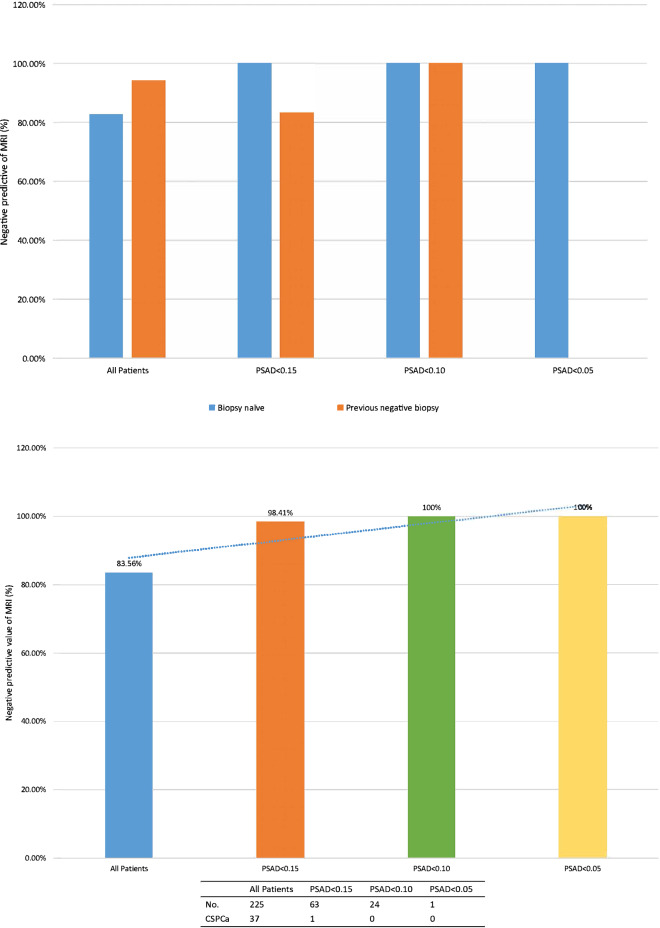
Negative predictive value of MRI for CSPCa. (**a**) by PSAD. (**b**) by PSAD and PBx and PBx status.

The negative predictive value (NPV) of bpMRI for CSPCa detection improved with decreasing PSAD (Table 3 and Fig. 4). The NPV of negative bpMRI increased from 83.56% in all patients to 98.41% in patients with PSAD < 0.15 ng/ml/cm^3^. There was evidently improvement in the NPV of bpMRI in biopsy naive patients from 76.16% in those with PSAD 0.15 ng/ml/cm^3^ or greater to 100% in those with PSAD less than 0.15 ng/ml/cm^3^ (P < 0.001). But there was mild change in the NPV of patients with prior negative biopsy from 100% in those with PSAD 0.15 ng/ml/cm^3^ or greater to 83.33% in those with PSAD less than 0.15 ng/ml/cm^3^ (*P* = 0.353).

## Discussion

In our single central study patients were brought into if there were no suspicious lesions on prebiopsy bpMRI as defined by PI-RADS v2.1 criteria. And, of the 1,012 patients in our data we identified this homogenous cohort of 225. According to this group we attempted to identify predictors of absent CSPCa to clarify the NPV of bpMRI when combined with other negative predictors of CSPCa^13^. As a matter of fact, MRI negative patients account for 27% to 44% of all patients with MRI before PB^14–16^.

To data at our center we have performed PBs if clinically showed by elevated PSA, suspicious DRE and suspected lesion on MRI. A total of 208 patients (92.44%) underwent naive biopsy and 17 patients (7.56%) with previous negative biopsy underwent prebiopsy. The CSPCa and PCa detection rates were 16.44% and 27.11%, corresponding to NPV of 83.56% and 72.89%, respectively. The results are consistent with recent literature and resemble to those reported by Hansen et al. using PSAD < 0.1 ng/ml/cm^3^^17^.

But, in most reports predictors of absent CSPCa on PB were not analyzed in patients with negative MRI. Some researches were underpowered and had only a few events, precluding further evaluation. Ahmed HU et al. did this multicentre, paired-cohort, confirmatory study to test diagnostic accuracy of mpMRI and TRUS-biopsy against a reference test. They evaluated 576 patients who underwent mpMRI and biopsy with saturation transperineal prostate biopsy as the reference standard^18^. 158 patients (27%) had negative mpMRI and NPV of CSPCa was 76%. But in this study, mpMRI was performed at 1.5 T strength and researchers did not assess predictors of absent CSPCa on biopsy in the case of negative MRI.

A retrospective study described that PCa and CSPCa diagnosis-free survival probabilities of 1,225 patients with negative mpMRI after 2 years of follow-up^19^. The 2-year PCa and CSPCa diagnosis-free survival rate were 84% and 95% in group A (659 biopsy naive patients) but in group B (596 prior negative biopsy patients) the rates were 96% and 96%, respectively. Finally, they concluded that biopsy should be recommended even after negative mpMRI, particularly young patients or patients with high or rising PSA^19^. These NPVs seem higher than those in our report. And the age of patients with MRI negative diagnosed with CSPCa is relatively large in our data.

Adding clinical information to mpMRI may help improve the NPV^20,21^. Washino et al. analyzed 288 patients who underwent biopsies, including 127 (44%) with negative MRI^13^. The multivariate analysis revealed that PI-RADS v2 score and PSAD were independent predictors for PCa and CSPCa. Biopsy naive patients were divided into 4 groups, including those with PSAD < 0.15, 0.15 to 0.29 , 0.30 to 0.44 and 0.45 ng/ml/cm^3^ or greater, respectively. Only 2 of the 51 patients with PSAD less than 0.15 ng/ml/cm^3^ had CSPCa. However, it is important to note the high median PSAD of 0.26 ng/ml/cm^3^ in this cohort and the fact that only 52% of patients underwent 3 T MRI with 1.5 T MRI performed in 49%.

Distler et al. biopsied 1040 patients who assessed MRI by PI-RADS v1^20^. 344 of them (33%) had no suspicious lesions on mpMRI. Patients were stratified into 3 PSAD groups, including < 0.07, 0.07 to 0.15 and > 0.15 ng/ml/cm^3^, respectively. When combined with PSAD = 0.15 ng/ml/cm^3^ and PSAD < 0.15 ng/ml/cm^3^, the NPV of mpMRI for CSPCa increased from 79 to 89%. When combining PSAD and mpMRI, about 20% of the biopsies seem unnecessary.

We used PSAD and DRE suspicious for PCa, and excluded PV from multivariable analysis to avoid confounding factors. According to several reports and as recommended by NCCN Guidelines, we used a PSAD threshold of 0.15 ng/ml/cm^3^ for analysis^13,20,22^. According to research conclusions, the study of 285 cases of radical prostatectomy (RP) specimens showed that PSAD was better than PSA and GS in predicting positive surgical margins, extra capsular disease, lymph node invasion and seminal vesicle invasion^23^. By contrast, PSAD < 0.15 ng/ml/cm^3^ with less than 3 mm PCa found in only one positive core in biopsy were predictors of CIPCa in RP samples^24^.

In other negative MRI markers of patients, including PSAD, might replenish value in identifying those necessary repeat biopsy^23^. The recent analysis found that mpMRI should be considered in patients with previous negative biopsy who have suspicion for PCa and are being considered for repeat PB^25^. Patients with a history of prior negative biopsy have lower PCa detection rate on repeat biopsy. Ploussard et al. compared the data of 617 patients who underwent repeat biopsy with a mean of follow-up 19 months^16^. The researchers compared 16.7%, 16.9% and 12.5% PCa detection rates for the 2nd, 3rd and 4th sets of repeat biopsies, respectively. They found that PSAD > 0.15 ng/ml/cm^3^ more than doubled PCa detection on repeat biopsy, too.

Most interestingly, in our study, only 1 patient had CSPCa on repeat biopsy when combining negative MRI, PSAD < 0.15 ng/ml/cm^3^ and previous negative PB history.

However, patients with PSAD ≥ 0.15 ng/ml/cm^3^, suspicious DRE and naive PB history should undergo PB even if bpMRI doesn’t show lesions (Table 1).

Our research was limited by a relatively small number of patients. The number of prior negative biopsies is relatively small compared to the number of naive biopsies. However, our group size is bigger than those in prior reports. The patients who underwent prior negative biopsies used different biopsy protocols with a varying number of cores taken, which were not available from reports done elsewhere. Nevertheless, we report the BMI of patients and further stratified analysis of PSAD but to our knowledge no prior publication has done so. According to PI-RADS criteria, good quality bpMRI were performed, interpreted and reported by experienced radiologists. And to reduce research errors, we asked our agency's most experienced uroradiologists to reevaluate MRI reports rigorously. This detail was rarely seen in reports elsewhere.

Our study was a single-institute study. A major limitation of it was relatively small sample size. In addition, there was a potential selection bias to this retrospective study. Therefore, more prospective studies with a larger cohort of subjects were needed to support the present findings.

Our data reflected real practice. PBs were performed by well-experienced urologists, and histological results were reviewed by a specialized pathologist.

## Conclusions

The NPV of bpMRI for CSPCa was 83.56%, which improved to 98.41% when combined with PSAD < 0.15 ng/ml/cm^3^. Patients with a combination of negative bpMRI and PSAD < 0.15 ng/ml/cm^3^ can securely avoid PB as only 1 patient with prior negative biopsy had CSPCa on PB. On the contrary, PB should be considered in patients regardless of negative bpMRI, particularly those with PSAD ≥ 0.15 ng/ml/cm^3^ and suspicious DRE.
